# How to Evaluate the Effectiveness of Health Promotion Actions Developed Through Youth-Centered Participatory Action Research

**DOI:** 10.1177/10901981211046533

**Published:** 2021-10-09

**Authors:** Manou Anselma, Teatske M. Altenburg, Jos W. R. Twisk, Xinhui Wang, Mai J. M. Chinapaw

**Affiliations:** 1 Amsterdam UMC, Vrije Universiteit Amsterdam, Department of Public and Occupational Health, Amsterdam Public Health research institute, Amsterdam, The Netherlands; 2 Amsterdam UMC, Vrije Universiteit Amsterdam, Department of Epidemiology and Biostatistics, Amsterdam Public Health research institute, Amsterdam, The Netherlands; 3 College of Computer Science, Qinghai Normal University, Xining, Qinghai, China

**Keywords:** EBRB, children, health behavior, participatory action research, controlled trial

## Abstract

Most actions targeting children’s health behaviors have limited involvement of children in the development, potentially contributing to disappointing effectiveness. Therefore, in the 3-year “Kids in Action” study, 9- to 12-year-old children from a lower-socioeconomic neighborhood were involved as coresearchers in the development, implementation, and evaluation of actions targeting health behaviors. The current study describes the controlled trial that evaluated the effects on children’s energy balance-related behaviors, physical fitness, and self-rated health, as well as experienced challenges and recommendations for future evaluations. Primary school children from the three highest grades of four intervention and four control schools were eligible for participation. Outcome measures assessed at baseline, and at 1- and 2-year follow-up were as follows: motor fitness by the MOPER test (*N* = 656, *N* = 485, *N* = 608, respectively), physical activity and sedentary behavior by accelerometry (*N* = 223, *N* = 149, *N* = 164, respectively), and consumption of sugar sweetened beverages and snacks and self-rated health by a questionnaire (*N* = 322, *N* = 281, *N* = 275, respectively). Mixed-model analyses were performed adjusted for clustering within schools and relevant confounders. Significant beneficial intervention effects were found on self-reported consumption of energy/sports drinks at T2 versus T0, and on total time and ≥5-minute bouts of moderate-to-vigorous physical activity at T1 versus T0. Significant adverse effects were found on “speed and agility” and “coordination and upper-limb speed.” No other significant effects were found. The inconsistent intervention effects may be explained by the dynamic cohort and suboptimal outcome measures. We advise future studies with a similar approach to apply alternative evaluation designs, such as the delayed baseline design.

## Impact Statement

That health behavior change is difficult is an understatement. Many interventions have been developed to improve children’s energy balance-related behaviors (EBRBs), but their effectiveness is mostly limited and of short duration. Participatory action research with children in which they cocreate actions may lead to more attractive, better tailored and thereby more effective interventions. The current study describes a controlled trial that evaluates a participatory action research together with 9- to 12-year-old children as coresearchers to develop, implement, and evaluate actions to improve children’s EBRBs. This article presents valuable lessons for designing future studies evaluating the effectiveness of health promotion actions cocreated by children in a participatory research process.

## Introduction

In the Netherlands, the number of children with overweight and obesity gradually declined in the past decade (Dutch Bureau for Statistics [Centraal Bureau voor de Statistiek–Dutch], 2019), also in the city of Amsterdam (City of Amsterdam, 2017). Despite this promising development, the number of children with overweight or obesity with a low-socioeconomic position (SEP; based on family income, household conditions, parental education, and occupation) or from a non-Western background remains high (City of Amsterdam, 2017; Franssen et al., 2015). These children are disproportionally affected by unhealthy behaviors and related health effects, for example, because healthy foods are not/cannot be prioritized, cultural habits, and limited finances (Anselma et al., 2018). Previous intervention studies showed that these groups are often not reached by existing health promotion programs (Bonevski et al., 2014; Craike et al., 2018), which could be due to unsuitable communication materials, communication channels, or divergent attitudes of academic researchers (Carroll et al., 2011; Harkins et al., 2010). Therefore, changing behaviors in children from low SEP environments remains a huge public health challenge.

EBRBs—that is, behaviors that effect energy intake or expenditure, such as physical activity, dietary behavior, and screen time—have been associated with overweight and obesity in children (Romieu et al., 2017; te Velde et al., 2012), with children from lower educated parents being more likely to engage in unhealthy EBRBs (Fernandez-Alvira et al., 2013). However, few interventions proved effective in improving EBRBs in children from low SEP environments, and those that are effective showed small effects (Anselma et al., 2020; Wijtzes et al., 2017). One explanation could be that intervention strategies are insufficiently tailored to children from low SEP environments and therefore the strategies do not match their personal and community’s context, culture, needs, and interests. Interventions that are specifically designed for, or even together with, children from these communities may better fit their needs and interests and thereby may be more effective (Anselma et al., 2020).

The “Kids in Action” study combined youth-centered participatory action research and intervention mapping, to structurally develop actions in collaboration with children from a low SEP neighborhood to improve their EBRBs (Anselma, Altenburg, Emke, et al., 2019). Participatory action research is increasingly being used in public health especially in so-called hard-to-reach communities, as this bottom-up approach could lead to, for example, a better understanding of the community, better tailored actions, positive community development, and empowerment (Anyon et al., 2018; Lems et al., 2020; Shamrova & Cummings, 2017). To improve EBRBs in children from low SEP environments and with that fight for health equity, the health promotion sector needs to adopt such approaches. Participatory action research with youth is a research approach in which children are trained as coresearchers and work side-by-side with researchers (Kellett, 2005; Langhout & Thomas, 2010; London et al., 2003). Children study their own environment and develop solutions for problems they identify. We combined this participatory approach with intervention mapping, which is a stepwise approach for identifying behavioral determinants and developing evidence-based strategies (Bartholomew Eldredge et al., 2016). We added intervention mapping to structure the action development process and stimulate use of evidence-based theoretical methods and strategies.

The process evaluation of the Kids in Action study showed that the cocreated actions were well received, both by the children and other community members (Anselma et al., 2020). Children and community partners mentioned that empowerment of children, who actively participated in the participatory action research, improved. Moreover, these children developed skills such as critical awareness and self-confidence as well as research skills. Community partners indicated that in children of the intervention schools awareness about EBRBs improved, but they questioned whether the actions also improved their actual behavior. Evidence for the effectiveness of applying participatory action research in the field of health promotion is currently lacking (Anyon et al., 2018; Jacquez et al., 2013). For example, effects of participatory developed actions on children’s EBRBs have rarely been evaluated in a controlled trial design. Challenges for the effect evaluation of participatory action research are, for example, that at the start it is unknown which specific behaviors will be targeted and therefore what optimal outcome measures are. Therefore, the current study describes the effect evaluation of the Kids in Action study on children’s dietary behavior, physical activity, sedentary behavior, physical fitness, and self-rated health, using a controlled design over the course of 3 years, and the experienced challenges and recommendation for future evaluations. Some challenges are highlighted in the methods sections, and elaborated on in the discussion.

## Method

### Kids in Action

The Medical Ethics Committee of the VU University Medical Center approved the study protocol (2016.366). Kids in Action was a 3-year participatory action research, taking place in a low SEP neighborhood in Amsterdam, The Netherlands. The neighborhood was characterized by high numbers of residents with a non-Western background (50%; Municipality of Amsterdam, 2017a) and high numbers of childhood overweight with 30% of the 10-year-olds having overweight or obesity in 2017–2018 (Municipal Health Services Amsterdam, n.d.). Moreover, in 2015–2016, 31% of children younger than the age of 18 years grew up in a household with an income up to 110% of the Dutch minimum standard and capital below the social welfare limit (Municipality of Amsterdam, 2017b). Participatory action research is mostly conducted in low SEP communities (Shamrova & Cummings, 2017), as these communities can benefit most from such an approach by becoming empowered, learning new skills, and developing actions suitable to their needs (Ozer, 2017). In Kids in Action, children participated in the development, implementation, and evaluation of actions, as explained in detail elsewhere (Anselma, Altenburg, Emke, et al., 2019). In Kids in Action, we collaborated with children through their schools. Schools were chosen as a setting because we wanted to collaborate with a diverse group of children that could benefit most from participating in our study. This would have been different when, for example, working together with a sports club (i.e., only children interested in sports) or after school day care (i.e., children of high-income families). Second, because of the close collaboration between the local municipality and the academic researchers, it was a setting that was well accessible. Third, schools gave us indirect access to parents and other community partners which could help the reach and impact of the developed actions.

**Figure 1.**
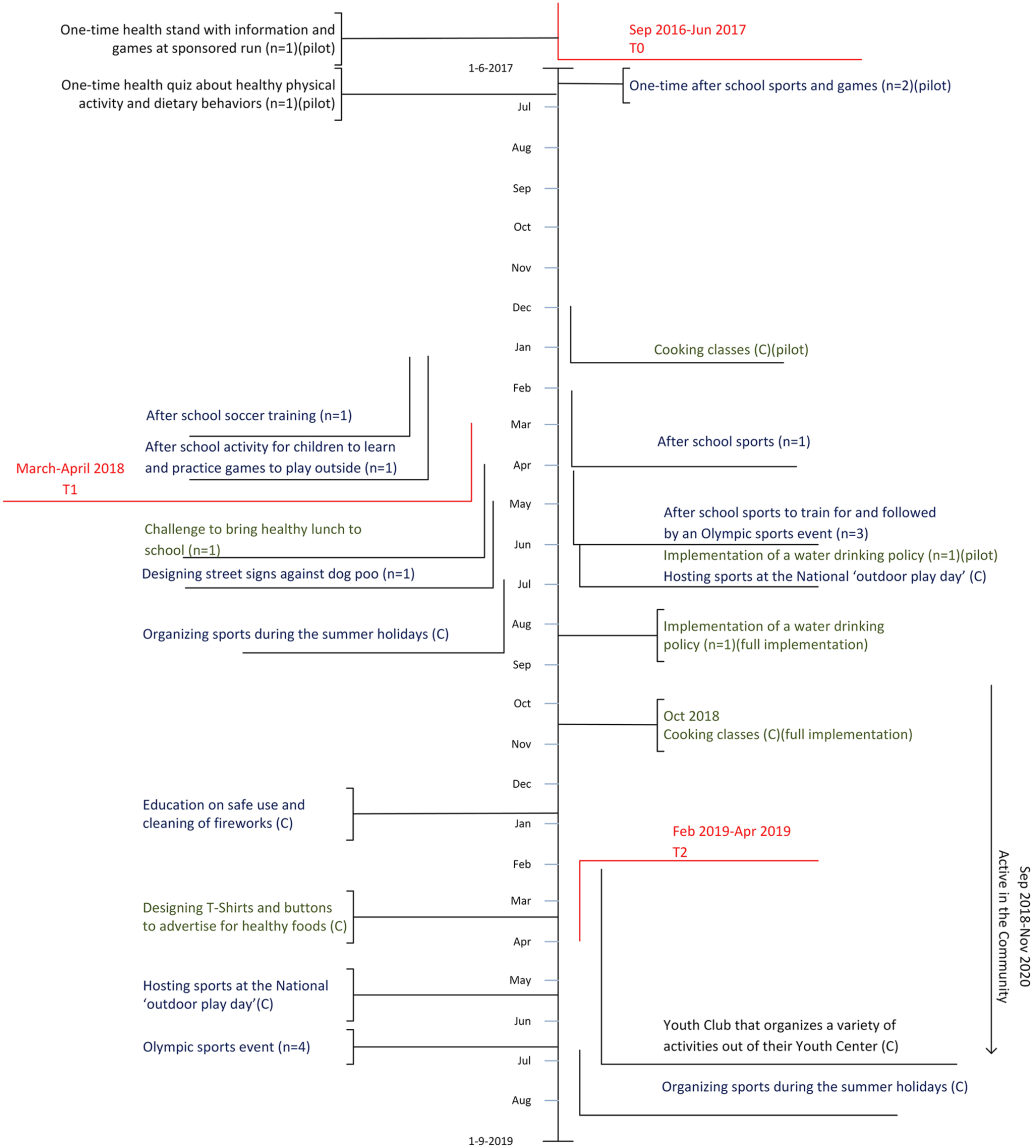
Timeline of implementation of cocreated actions. *Note.* The vertical length of the lines represent the duration of the actions. (*n*) = number of schools involved; (C) = community activity; blue = promoting healthy physical activity; green = promoting healthy dietary behavior; black = promoting healthy physical activity and dietary behavior.

In the first year of this study, the health needs of children were identified in a participatory needs assessment (Anselma et al., 2018). This needs assessment resulted in the focus on improving children’s physical activity and dietary behavior. In the first 2 years a participatory group was installed in each of the four intervention schools. These so called “Action Teams,” consisting of 6 to 8 children aged 9 to 12 years old and a facilitating academic researcher, developed, implemented, and evaluated actions (Anselma, Altenburg, Emke, et al., 2019). In the third year, one Action Team was established with representatives of three schools and they again worked together on new actions to be implemented in their neighborhood. Children could self-subscribe for the Action Teams and some children were specifically suggested by the teachers because teachers thought those children would like to participate and could miss school lessons. The meetings lasted 45 to 60 minutes and depending on the schools occurred during or after school hours. The meetings were semistructured, starting with a short game and introduction, and ending with a reflection and a game. For the content of the meetings, we followed a general outline based on the intervention mapping protocol, but we were flexible for whatever came to the table as not all children were always present due to, for example, birthday parties or children were distracted with something that happened during the day and wanted to share that. In the first few meetings, more time was spent on getting to know each other and learning research skills. Next, children conducted and analyzed their own research, intertwined with related skill development exercises. From the results of their research the Action Teams identified the most important problems and barriers children faced for engaging in healthy behaviors. The Action Teams came up with ideas for how to improve the situation, which were linked to and strengthened by evidence-based strategies identified by academic researchers. For the best ideas implementation plans were made together with relevant community partners, who helped implement the actions. The actions varied in reach (e.g., a one-time health stand at a sponsored run at one school, an Olympic sports event for four schools) and required resources (e.g., a few items for a health stand versus materials and finances for an Olympic sports event for 350 children). The implemented actions are depicted in Figure 1.

### Study Design

All four primary schools in the intervention neighborhood were approached by the local government and invited to participate as intervention schools. Within these intervention schools, the Actions Teams developed and implemented actions for children of the three highest grades of their school. As mentioned in Textbox 1, control schools were recruited from neighborhoods with inhabitants with similar socioeconomic characteristics. Schools in these neighborhoods were contacted (*N* = 22) until four schools agreed to participate. Control schools did not partake in the participatory design of action development, but only participated in the measurements. The four control schools and four intervention schools participated in three measurement waves as part of the controlled trial. The first wave took place throughout the school year 2016–2017 and was considered the baseline (T0). In the school year 2017–2018, measurements were conducted in March–April 2018 (T1). The last measurement wave was conducted in February–April 2019 (T2). Each year all children in the three highest grades of the schools were invited to participate in the measurements. This resulted in a dynamic cohort, where some children were invited for two or three measurements, others only for one (e.g., children in the highest grade in the first year of the study only participated once). Thus, the number of measurements varies per age group.

**Textbox 1.** 

Challenge 1—study design We chose for a controlled design as we wanted a robust design to evaluate our approach aimed at improving children’s EBRBs. Applying a controlled design for evaluating a participatory approach brings about two important challenges. The first challenge is the selection of adequate control schools. We focused our participatory approach on all schools in one particular community, making randomization impossible. Instead, we selected control schools from similar communities based on percentage of children with overweight, cultural diversity, and family income. The second challenge is having a dynamic cohort. We chose for a school-based study including children from Grades 6 to 8, that is, 9- to 12-year-old children. As children change grades and after Grade 8 leave school, we had a dynamic cohort, resulting in many missing data.

**Textbox 2.** 

Challenge 2—measurements Due to the participatory approach the exact focus of the cocreated actions was unknown at the start, complicating the choice of optimal outcome measures. We knew from the needs assessment that the focus would be on physical activity and dietary behaviors, but within those areas the actions could still be targeting various subbehaviors and determinants. Therefore, we chose to measure a variety of subbehaviors and determinants of physical activity and dietary behaviors with existing tools, as well as two more distal outcome measures: neuromotor fitness and self-perceived health.

### Procedures

**Figure 2.**
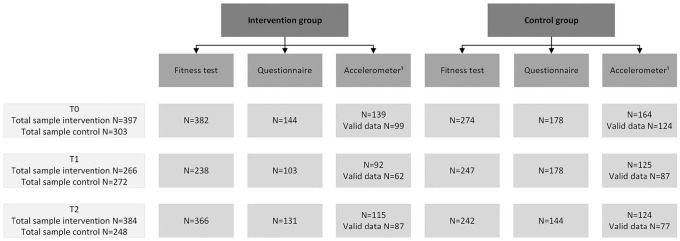
Flowchart of participants in the Kids in Action effect measurements. *Note.* Each year, children of Grades 6/7/8 participated in the measurements, resulting in a different sample at each time point. ^1^Participants needed a minimum of 8 hours of wear time per day on at least 4 days, including at least one weekend day, to be included in the analyses.

The Motor Performance (MOPER) fitness test was included to measure neuromotor fitness, a self-report questionnaire to assess self-perceived health, sports participation, outdoor play, sedentary behavior, consumption of sugar sweetened beverages and high-energy snacks, and accelerometers for physical activity and sedentary behavior. The MOPER fitness test was part of the school curriculum for children in the three highest grades and data were collected anonymously. Parents received an information letter from the physical education teacher. Attached was a refusal form to be signed and returned if they did not approve of MOPER fitness test results to be anonymously shared with the academic researchers. For the accelerometer and self-report questionnaire, each year children of the three highest grades received an information letter with attached an informed consent form that at least one parent had to sign to approve participation in the measurements. Parents could contact the academic researchers by phone or email in case they had questions or wanted more information. Because of the different consent procedures, the number of participants varied between the MOPER, questionnaire, and accelerometer, as depicted in Figure 2. We calculated that 240 children in the intervention group and 240 in the control group are needed to detect a difference of 0.15 *SD* in the aforementioned outcome variables (Anselma, Altenburg, & Chinapaw, 2019).

#### MOPER Fitness Test

The MOPER fitness test consists of eight test items: 10 × 5 meter run, leg-lifting while laying down, plate-tapping, bent-arm hang, sit-and-reach, arm-pull, standing high jump, and a 6 minutes run test. The MOPER fitness test items have shown acceptable validity and reliability for estimating neuromotor fitness in 9- to 12-year-old children (Leyten, 1982). For practical reasons, the hand-grip test was used instead of the arm-pull test and the 6-minute run test was omitted. The hand-grip test has also shown acceptable validity for measuring children’s arm strength (Gasior et al., 2020). Seven test items were included measuring speed and agility, strength, flexibility, and coordination and upper-limb speed (see Supplemental 1). The MOPER fitness test was administered during physical education by the physical education teacher with assistance of academic researchers or sports instructors. The class was divided in seven groups who completed all test items in the same order. Tests were conducted barefoot to limit (dis)advantage of different footwear.

#### Questionnaire

The questionnaire was developed based on validated items from the ENERGY-child questionnaire (Singh et al., 2011), the DOiT questionnaire (Janssen et al., 2014), and the EuroQol (Ravens-Sieberer et al., 2010), that covered identified determinants of overweight in the needs assessment (Anselma et al., 2018). The developed questionnaire consisted of nine sections: (a) Demographic and Family characteristics, (b) Soft drinks consumption, (c) Energy and sport drinks consumption, (d) Sweets consumption, (e) Snack consumption, (f) Playing outdoor, (g) Sports participation, (h) Screen viewing behavior, and (i) Perceived health (Anselma, Altenburg, & Chinapaw, 2019). Participants completed the questionnaire during school hours under the supervision of two trained academic researchers and the class teacher. Each section was explained by the academic researcher before that part of the questionnaire was collectively filled in. During the completion of the questionnaire, children were free to ask questions or withdraw from participation at any time. Children needed approximately 45 minutes to complete the questionnaire.

If possible, categorical variables were recoded into continuous variables. For example, the frequency of soda consumption was multiplied with the sum of number of glasses, cans, and bottles of soda consumed. Covariates were as follows: gender, birth country of parents, having younger/older siblings, living with both parents or otherwise, and speaking mainly Dutch at home or not.

#### Accelerometer

Time spent in physical activity and sedentary behavior was assessed using the Actigraph accelerometer. Children were asked to wear the accelerometer on their right hip for seven consecutive days during waking hours, with the exception of water activities and heavy contact sports. The Actigraph was set on a sample frequency of 100 Hz and data were analyzed in 15-second epochs between 07.00 a.m. and 10.00 p.m. (Chinapaw et al., 2014). Nonwear time was defined as a period of at least 60 consecutive minutes of zero counts (Chinapaw et al., 2014). For inclusion in the data analysis, each participant needed at least 4 days with a minimum of 8 hours wear time per day, including at least one weekend day.

Accelerometer count data were processed using a custom-made program developed in R. A cut point of ≤25 counts per 15 seconds (counts/15-sec) was selected for sedentary behavior (Fischer et al., 2012; Trost et al., 2011), 26 to 573 counts/15-sec for light physical activity and ≥574 counts/15-sec for moderate-to-vigorous physical activity (MVPA; Evenson et al., 2008). A sedentary bout was defined as a period of at least 10 consecutive minutes <25 counts/15-sec. An MVPA bout was defined as a period of at least five consecutive minutes ≥574 counts/15-sec with 10% tolerance allowed below the threshold and an absolute tolerance of three consecutive minutes.

**Textbox 3.** 

Challenge 3—analyses Challenges regarding the analyses were the many missings in the data due to low participation rates and the dynamic cohort. Furthermore, not all children who participated in the action development or in the implemented actions, participated in the measurements. Also, not all actions were accessible to all children, nor was participation in actions consistently registered. This creates the challenge of linking the participatory approach to the health behaviors outcomes.

### Analyses

Means (x̅) and standard deviations (*SD*) or medians (x~; in case of normally distributed variables) and interquartile ranges (25th–75th percentiles; in case of skewed variables) were calculated for descriptive purposes. For all regression analyses, the residuals of linear regression analyses were used to check the assumptions of normality and homoscedasticity. Linear mixed-model analyses with a four-level structure (i.e., repeated measures were clustered within children, children were clustered within classes and classes were clustered within schools) were used to examine the difference in the outcome variables between the control and the intervention group for the questionnaire and accelerometer data. For the MOPER fitness data, a three-level structure was used because the data were collected anonymously. Linear mixed-model analyses was applied as these analyses adequately deal with missing data (Twisk et al., 2018). There was a substantial amount of missing data by design in the present study because data was collected of children in Grades 6/7/8 for 3 years, instead of following the same group of children for 3 years. The linear mixed-model analyses included time (represented by two dummy variables) and the interaction between group and time. The latter indicated the difference in outcome between the groups at the two follow-up moments (Twisk et al., 2018).

Analyses using MOPER fitness test data were adjusted for gender and age. Analyses using questionnaire and accelerometer data were adjusted for ethnicity and living with both parents. Analyses using the accelerometer data were further adjusted for wear time. For all analyses, betas and 95% confidence intervals (CIs) were calculated. The statistical analyses were conducted using IBM SPSS Statistics 24.0.

In case assumptions of normality and homoscedasticity were not met, log-transformations were conducted. The variables “bent-arm hang” of the MOPER fitness test, “consumption of sodas” and “consumption of energy and sports drinks” of the questionnaire, and “MVPA accumulated in bouts ≥5 minutes” of the accelerometer, had a skewed distribution with an excess of zeros and were therefore analyzed using tobit mixed models analyses. Tobit mixed models analyses were performed in STATA (version 15).

## Results and Discussion

Figure 2 presents the flowchart of participants in the measurements. Supplemental 2 provides the characteristics of children participating in the MOPER fitness test. Participating children were equally divided across grades with a mean age of 10.6 years old and 47% to 55% of the participating children were girls. Supplemental 3 and Supplemental 4 present the characteristics of the subgroup of children who completed the questionnaire and had valid accelerometer data, respectively. More girls than boys participated in these measurements (57%–71%). In this subgroup, a substantial number of children had parents who were born in Morocco or Turkey (27%–41%), or in another country than the Netherlands (29%–38%). Most children spoke Dutch at home (72%–91%), lived with both parents (67%–85%) and had siblings (84%–93%).

Table 1 provides the results of the mixed model analyses. The actions had significant adverse effects on the “10 × 5 meter run” (β = 0.5 sec, 95% CI [0.0, 1.0]) at T2 versus T0 and “plate-tapping” (β = 0.5 sec, 95% CI [0.1, 0.9]) at T1 versus T0, the latter due to improved scores in the control group. We found a significant beneficial intervention effect on consumption of energy/sports drinks at T2 versus T0 (β = −1023.1 mL, 95% CI [−1940.7, −105.5]) due to an increase in the control group. Based on the accelerometer data, the intervention had significant beneficial effects at T1 versus T0 on total MVPA (β = 9.5 min per day, 95% CI [2.5, 16.5]) and MVPA in bouts (β = 2.0 minutes per day, 95% CI [0.0, 3.9]). These effects were not present at T2 versus T0, due to an improvement in MVPA in the control group and a decline in the intervention group. No other significant effects were found.

**Table 1.** Effects (β [95% CI]) of Kids in Action on Neuromotor Fitness, Dietary Behavior, Physical Activity, Sedentary Behavior, and Self-rated Health.

	T1 vs. T0	T2 vs. T0
*MOPER* ^a^		
Bent-arm hang^b^ (s) ↑^c^	1.5 [−0.7, 3.7]	−0.7 [−2.7, 1.2]
10 x 5 meter run (s) ↓^d^	−0.4 [−0.9, 0.2]	0.5 [0.0, 1.0]*
Leg-lift^e^ (s) ↓	1.0 [1.0, 1.0]	1.0 [1.0, 1.0]
Plate-tapping (s) ↓	0.5 [0.1, 0.9]*	0.2 [−0.2, 0.6]
Sit-and-reach (cm) ↑	0.6 [−0.7, 2.0]	1.1 [−0.1, 2.3]
Hand-grip strength (kg) ↑	0.3 [−0.6, 1.2]	0.1 [−0.7, 1.0]
High-jump (cm) ↑	0.9 [−0.6, 2.3]	0.6 [−0.8, 1.9]
*Self-report* ^f^		
Consumption soda^b^ (mL/week)	−578.1 [−1798.0, 641.8]	−736.4 [−1910.9, 438.1]
Consumption energy/sports drinks^b^ (mL/week)	−192.4 [−1156.7, 771.8]	−1023.1 [−1940.7, −105.5]*
Consumption candy^e^ (portions/week)	1.0 [0.9, 1.1]	1.0 [0.9, 1.1]
Consumption snacks^e^ (portions/week)	0.9 [0.8, 1.1]	1.0 [0.9, 1.1]
Active transport to school (min)	−1.0 [−2.4, 0.5]	−1.5 [−3.0, 0.0]
Outside play (min/day)	3.6 [−15.0, 22.2]	−3.6 [−22.3, 15.1]
Sports participation^e^ (min/day)	1.0 [0.9, 1.1]	1.0 [0.9, 1.1]
Watching TV/movies (min/day)	6.1 [−15.4, 27.7]	−0.7 [−21.6, 20.3]
Gaming (min/day)	19.8 [−6.1, 45.8]	−0.9 [−26.1, 24.3]
Self-rated health (scale 0–100)	4.2 [−1.6, 9.9]	−1.4 [−6.9, 4.1]
*Accelerometer* ^g^		
Time spent sedentary (min/day)	−0.3 [−18.8, 18.2]	18.4 [−0.2, 37.1]
Time spent in LPA (min/day)	−6.2 [−24.2, 11.7]	−5.3 [−22.4, 11.7]
Time spent in MVPA (min/day)	9.5 [2.5, 16.5]**	−6.4 [−13.3, 0.5]
MVPA accumulated in bouts ≥5 min^b^ (min/day)	2.0 [0.0, 3.9]*	−1.0 [−2.9, 0.9]
Sedentary time accumulated in bouts ≥10 min (min/day)	−1.0 [−18.1, 16.2]	3.5 [−12.7, 19.8]

*Note.* Control group is coded as 0. LPA = light physical activity; MVPA = moderate-to-vigorous physical activity.

* *p* < .05. ^**^p < .01.

a Adjusted for *age* and *gender*. ^b^Tobit mixed models analysis performed. ^c^A higher value indicates a better test score. ^d^A lower value indicates a better test score. ^e^Data log-transformed, β has to be interpreted as a ratio. ^f^Adjusted for *ethnicity* and *living with both parents*. ^g^Adjusted for *ethnicity, living with both parents* and *wear time*.

### Challenge 1 and Recommendations—Study Design

We recruited four control schools from neighborhoods with similar characteristics as the intervention schools. However, the control schools also had certain policies targeting healthy behaviors, possibly diluting intervention effects. The favorable intervention effects on the consumption of energy/sports drinks at T2 versus T0 resulted from an increase in the consumption of energy/sports drinks of children in the control group. Promotion of drinking water was implemented as part of “usual care” by community organizations and local government in both intervention and control neighborhoods and in most schools. Additionally, one of the cocreated actions promoted drinking water and raised awareness on sugar-sweetened beverages. These child-initiated actions within Kids in Action at intervention schools could have contributed to the stabilization of consumption of energy/sports drinks in the intervention group, versus an increase in the control group. This is supported by the process evaluation, which showed that Kids in Action stimulated organizations in the intervention neighborhood to prioritize healthy lifestyle policies (Anselma et al., 2020). Since we did not know the focus of actions at the start, we did not measure consumption of water (see Challenge 2).

We recommend future participatory studies to apply more flexible study designs to deal with some of the challenges such as finding suitable control schools, monitoring what policies are being implemented at those schools, and the varying sample throughout the study. An example of a more flexible design is the extended cohorts design, as in this design time point one of the study sample serves as a baseline for age-equivalent groups at following time points (Olweus, 2005).

### Challenge 2 and Recommendations—Measurements

An intricate and inevitable challenge of evaluating participatory studies is that beforehand it is unknown what behaviors will specifically be targeted by the developed actions (Anyon et al., 2018). Consequently, it is unknown at the start what specific outcome measures are optimal. For example, in the present study, a water policy was successfully implemented at one school, but water consumption was not measured. Also, the adverse effects on some fitness items are difficult to explain, but since no actions were developed that specifically targeted neuromotor fitness, these could be chance findings. Future participatory studies might add delayed baseline measurements to include measures of outcomes that were unknown at baseline. Additionally, process evaluations are of utmost importance to provide insight into the participatory process, community experiences and how these may have influenced the targeted health behaviors (Lindquist-Grantz & Abraczinskas, 2018).

It is difficult to compare the current study with previous studies as to the best of our knowledge there are no other participatory studies aimed at improving children’s EBRBs with a similar level of child participation throughout the development, implementation, and evaluation of actions, and including a controlled trial design. Looking more generally to previous studies evaluating interventions developed in participation with children or adolescents aiming to improve EBRBs, these interventions also showed small or inconsistent effects (Frerichs et al., 2016; Froberg et al., 2018; Verloigne et al., 2017), similar to interventions which did not include participatory methods (Kornet-van der Aa et al., 2017; Metcalf et al., 2012; Olstad et al., 2017). Participatory action research with children does show promising results in creating actions that adhere to children’s needs and interests, community engagement, improving children’s awareness of unhealthy behavior, and developing several valuable life skills (Anselma et al., 2020; Anyon et al., 2018; Shamrova & Cummings, 2017). Therefore, we hope that future studies aiming to improve children’s EBRBs apply the lessons learned from studies such as ours, and further examine how effectiveness of cocreated interventions in participatory action research can be properly evaluated and improved.

### Challenge 3 and Recommendations—Analyses

We want to acknowledge that the design of this study and the analyses have their limitations. As academic researchers, we are however obliged to use and report on the data that we have, as the participants have dedicated their time and efforts (Alley et al., 2015; World Medical Association, 2018). We looked for analyses that best fitted our data and chose linear mixed model analysis as this adequately handles missing data. For future participatory studies that want to include a controlled design, we have the following recommendations. First, it is recommended to clearly register the children who participate in actions and action development to enable including this in the analyses. For example, by registering attendees to sessions and events, retrospectively asking children their exposure/attendance/involvement or incorporating monitoring of dose/response in the process evaluation using a meaning for “dose” that fits the study (Rowbotham et al., 2019). This will help in gaining knowledge on the effectiveness on EBRBs of participatory approaches and the actions it produces. Our second recommendation is to ensure that you have considerable time and resources for recruitment of participants. We did not reach the required sample size, and were therefore underpowered for detecting intervention effects. Recruiting participants, especially in lower-socioeconomic areas, can be challenging, but it is not impossible when using the right approaches (Carroll et al., 2011; Harkins et al., 2010). For example, working together with local organizations who are already known by the children and their parents, using informal networks and develop recruitment materials together with the local community so that they match their cultures, interests, and their level of understanding. Last, we recommend academic researchers to be creative in working with their data. An example is to create hypotheses that match the data set and relate to the implemented actions, before analyzing the data. For example, in Kids in Action a water policy was implemented at one school, so a hypotheses would be that water consumption of children would have increased more at that school compared with the other intervention schools. This leads to more tailored analyses than just comparing intervention groups with control groups.

### Recommendations—Participatory Approach

In Kids in Action the focus was on the collaboration with children (Anselma, Altenburg, Emke, et al., 2019). This process was optimized by closely collaborating with schools, community organizations, and the local government. By developing and implementing actions with them, Kids in Action hoped to also reach changes in the system and local/organizational policies. However, not all partners were engaged in all phases of the project and most actions focused on the school and neighborhood environment and less on the home environment and parents (Anselma et al., 2020). Ecological models describe that when aiming to improve EBRBs in children, the system surrounding the child needs to be targeted (Davison & Birch, 2001; Lytle, 2009). We recommend future participatory studies to obtain a systems approach, involving important stakeholders on all system levels and thereby develop synergistic actions, and also evaluate their impact on different levels and with all involved stakeholders (Frerichs et al., 2016; Gates, 2016; Waterlander et al., 2020). Additionally, in participatory research with children, children decide to work on a topic that is relevant to them and that they want to address (London et al., 2003; Ozer, 2017). For children, this may mean that they do not wish to participate in all topics related to a healthy lifestyle and perhaps even decline some power. In Kids in Action, children mainly developed actions related to sports and play, and were less interested in developing actions to improve their dietary behavior. This could explain the favorable effects on total MVPA and MVPA in bouts (Anselma, Altenburg, Emke, et al., 2019). Future studies could discuss with the children which topics they would like to address themselves and which topics they rather leave to others (e.g., researchers, parents, and teachers). Therefore, we also recommend future studies to discuss with children their desired level of power sharing on each of the research topics, to make sure all topics are covered and children participate on the level of their choosing (Hart, 1992; Wong et al., 2010). Last, although the duration of Kids in Action was 3 school years few children could actively participate in the development, implementation, and evaluation of actions. In Kids in Action, we closely collaborated with 13 to 25 children per year, over the course of 3 years, and the majority of actions were developed and implemented in the second year (Anselma, Altenburg, Emke, et al., 2019; Anselma et al., 2020). Our process evaluation indicated that community partners put healthy behaviors and child participation higher on their agenda, that professionals from different organizations worked more closely together, that children’s awareness about healthy behaviors improved, as well as children’s empowerment (Anselma et al., 2020). However, it may take more time and participation of more children, parents, and other stakeholders for these improvements to result in detectible changes in EBRBs (Anselma et al., 2020; Moore et al., 2019). We recommend future studies to aim for structural changes in policy and practice, as we believe that participation of children in decision making and the cocreation of actions has many benefits and therefore should be embedded in for example the education of teachers and social workers.

### Strengths and Limitations

The current study has several strengths and limitations. A limitation of this study is the low participation rate in the self-report questionnaire and accelerometer data, limiting the power of our study sample. Additionally, the actions have reached a limited number of children while the evaluation also included children who did not participate in certain actions, for example, because an action was not implemented at their school. Relatedly, we did not register which children participated in which actions, so we did not have any information about the intervention dose received per child. Another limitation is that no valid and reliable questionnaires on the consumption of sugar-sweetened beverages and unhealthy snacks, and sports and outdoor play participation were available. So even though our questionnaire consisted of the most valid and reliable items from existing questionnaires, the questionnaire may have been inadequate in detecting subtle changes in EBRBs. Last, a limitation is the dynamic cohort, making it impossible to draw strong conclusions about the intervention effect. This is further impeded by the choice of one intervention school to withdraw from participation in the second year.

An important strength of this study is that it included a community approach, in which all primary schools in the community participated, as well as the local government and relevant stakeholders. A second strength is that this study assessed actual behavior change both in interventions and control schools, which rarely occurs in participatory action research (Anyon et al., 2018; Jacquez et al., 2013). Furthermore, intervention and control schools were similar regarding childhood overweight, ethnicity, and socioeconomic status. Future studies could consider a three-arm study adding a treatment arm where actions are developed and implemented top–down without child participation to examine the added effect of child participation.

## Conclusion

In the Kids in Action study, 9- to 12-year-old children cocreated actions to promote physical activity and healthy dietary behaviors in peers using a participatory approach. Despite positive findings on children’s empowerment and awareness of healthy behaviors observed in the process evaluation (Anselma et al., 2020), the current effect evaluation showed no consistent beneficial effects on children’s physical activity, sedentary behavior, dietary behavior, neuromotor fitness and self-perceived health. To obtain larger effects, we recommend future participatory action research to collaborate with more children and more intensively with school staff, families, and local organizations, trying to create effects in the larger system surrounding the child. To measure the effects more accurately, we recommend alternative evaluation designs such as the extended cohorts design. Additionally, we advocate for the value of process evaluations in participatory action research with youth, obtaining stakeholders’ experiences as well as including relevant effect measures from the stakeholder perspective.

## Supplemental Material

sj-docx-1-heb-10.1177_10901981211046533 – Supplemental material for How to Evaluate the Effectiveness of Health Promotion Actions Developed Through Youth-Centered Participatory Action Research Click here for additional data file. Supplemental material, sj-docx-1-heb-10.1177_10901981211046533 for How to Evaluate the Effectiveness of Health Promotion Actions Developed Through Youth-Centered Participatory Action Research by Manou Anselma, Teatske M. Altenburg, Jos W. R. Twisk, dr. Xinhui Wang and Mai J. M. Chinapaw in Health Education & Behavior

sj-docx-2-heb-10.1177_10901981211046533 – Supplemental material for How to Evaluate the Effectiveness of Health Promotion Actions Developed Through Youth-Centered Participatory Action Research Click here for additional data file. Supplemental material, sj-docx-2-heb-10.1177_10901981211046533 for How to Evaluate the Effectiveness of Health Promotion Actions Developed Through Youth-Centered Participatory Action Research by Manou Anselma, Teatske M. Altenburg, Jos W. R. Twisk, dr. Xinhui Wang and Mai J. M. Chinapaw in Health Education & Behavior

sj-docx-3-heb-10.1177_10901981211046533 – Supplemental material for How to Evaluate the Effectiveness of Health Promotion Actions Developed Through Youth-Centered Participatory Action Research Click here for additional data file. Supplemental material, sj-docx-3-heb-10.1177_10901981211046533 for How to Evaluate the Effectiveness of Health Promotion Actions Developed Through Youth-Centered Participatory Action Research by Manou Anselma, Teatske M. Altenburg, Jos W. R. Twisk, dr. Xinhui Wang and Mai J. M. Chinapaw in Health Education & Behavior

sj-docx-4-heb-10.1177_10901981211046533 – Supplemental material for How to Evaluate the Effectiveness of Health Promotion Actions Developed Through Youth-Centered Participatory Action Research Click here for additional data file. Supplemental material, sj-docx-4-heb-10.1177_10901981211046533 for How to Evaluate the Effectiveness of Health Promotion Actions Developed Through Youth-Centered Participatory Action Research by Manou Anselma, Teatske M. Altenburg, Jos W. R. Twisk, dr. Xinhui Wang and Mai J. M. Chinapaw in Health Education & Behavior
