# Supplementary material for: How to Evaluate the Effectiveness of Health Promotion Actions Developed Through Youth-Centered Participatory Action Research
Source: Health Educ Behav. 2021 Oct 9;50(2):199–210. doi: 10.1177/10901981211046533 (PMC10021122; doi:10.1177/10901981211046533)
Supplement: sj-docx-1-heb-10.1177_10901981211046533 – Supplemental material for How to Evaluate the Effectiveness of Health Promotion Actions Developed Through Youth-Centered Participatory Action Research [file sj-docx-1-heb-10.1177_10901981211046533.docx]

**Online supp 1:** Description of the MOPER fitness test items used in this study.

|  | Item | Characteristic | Description | Unit |
| --- | --- | --- | --- | --- |
|  | **10x5 meter run** | Speed and agility | 10 times running between 2 lines with a five meters distance as fast as possible, 2 attempts | s |
|  | **Plate tapping with one hand** | Coordination and upper limb speed | Tapping two plates alternately with the dominant hand 50 times as fast as possible, 2 attempts | s |
|  | **Bent-arm hang** | Upper body strength | Hanging from a horizontal bar with bended arms as long as possible, 1 attempt | s |
|  | **Hand-grip** | Hand-grip strength | Stand and hold a hand dynamometer in the dominant hand with a straight arm down. Pinch as hard as possible for two seconds. | kg |
|  | **Standing high jump** | Explosive leg strength | Jumping up from a standing position as high as possible, 2 attempts | cm |
|  | **Leg lifting while laying down** | Trunk and leg strength | Lifting outstretched legs 10 times while laying on back as fast as possible, 1 attempt | s |
|  | **Sit-and-reach** | Trunk and hamstrings flexibility | Reaching from sitting position with outstretched legs and arms as far as possible, 3 attempts | cm |

*Notes*. cm=centimeters, kg=kilograms, s=seconds.
