# Supplementary material for: How to Evaluate the Effectiveness of Health Promotion Actions Developed Through Youth-Centered Participatory Action Research
Source: Health Educ Behav. 2021 Oct 9;50(2):199–210. doi: 10.1177/10901981211046533 (PMC10021122; doi:10.1177/10901981211046533)
Supplement: sj-docx-2-heb-10.1177_10901981211046533 – Supplemental material for How to Evaluate the Effectiveness of Health Promotion Actions Developed Through Youth-Centered Participatory Action Research [file sj-docx-2-heb-10.1177_10901981211046533.docx]

**Online supp 2:** Characteristics of children who completed the MOPER fitness test.

|  | **T0** | | **T1** | | **T2** | |
| --- | --- | --- | --- | --- | --- | --- |
|  | **Intervention** N=382 | **Control**  N=274 | **Intervention**  N=238 | **Control**  N=247 | **Intervention** N=366 | **Control**  N=242 |
| **Grade**^1^ (%) 6  7  8 | 33.2  32.5  34.3 | 33.7  31.9  34.4 | 34.9  27.7  37.4 | 37.7  30.4  32.0 | 35.3  38.1  26.6 | 39.0  29.9  31.1 |
| **Females** (%) | 52.9 | 50.9 | 49.8 | 54.9 | 46.6 | 50.4 |
| **Mean age** (years) (SD) | 10.6 (1.0) | 10.6 (1.1) | 10.6 (1.1) | 10.5 (1.1) | 10.5 (1.0) | 10.5 (1.1) |
| **Bent-arm hang** ↑ (s) x͂ (IQR) | 2.0 (0.0-8.3) | 3.0 (0.0-8.0) | 3.0 (0.0-9.0) | 2.0 (0.0-6.0) | 3.0 (0.0-9.0) | 4.0 (0.0-9.0) |
| **10x5 meter run** ↓  (s) x̅ (SD) | 21.0 (2.1) | 21.3 (2.2) | 21.6 (2.7) | 21.7 (2.3) | 21.6 (2.5) | 21.4 (2.3) |
| **Leg-lift** ↓  (s) x͂ (IQR) | 15.3 (13.5-17.4) | 15.2 (13.4-17.3) | 17.0 (14.4-21.5) | 16.5 (14.3-20.0) | 15.5 (13.4-18.8) | 14.6 (12.9-17.5) |
| **Plate-tapping** ↓  (s) x̅ (SD) | 14.3 (1.9) | 14.9 (2.0) | 14.2 (2.2) | 14.3 (2.1) | 13.8 (1.9) | 14.1 (2.3) |
| **Sit-and-reach** ↑ (cm) x̅ (SD) | 27.6 (7.6) | 27.7(7.3) | 28.1 (7.7) | 27.5 (7.1) | 28.9 (7.5) | 28.0 (7.0) |
| **Hand-grip** (kg) ↑ x̅ (SD) | 21.6 (5.0) | 21.3 (4.8) | 21.8 (5.1) | 21.0 (5.3) | 21.2 (4.9) | 21.2 (5.0) |
| **High-jump** (cm) x̅ (SD) ↑ | 36.4 (7.4) | 35.9 (7.1) | 34.0 (6.2) | 32.3 (6.2) | 33.4 (6.7) | 32.3 (6.4) |

*Notes.* s=seconds, cm=centimeters, kg=kilograms, ↑=a higher value indicates a better test score, ↓=a lower value indicates a better test score. ^1^ Mixed grades (such as 6/7) are added to the lowest grades.
