# Supplementary material for: How to Evaluate the Effectiveness of Health Promotion Actions Developed Through Youth-Centered Participatory Action Research
Source: Health Educ Behav. 2021 Oct 9;50(2):199–210. doi: 10.1177/10901981211046533 (PMC10021122; doi:10.1177/10901981211046533)
Supplement: sj-docx-3-heb-10.1177_10901981211046533 – Supplemental material for How to Evaluate the Effectiveness of Health Promotion Actions Developed Through Youth-Centered Participatory Action Research [file sj-docx-3-heb-10.1177_10901981211046533.docx]

**Online supp 3:** Characteristics of children with self-report data on physical activity, screen behavior, dietary behavior and self-rated health.

|  | **T0** | | **T1** | | **T2** | |
| --- | --- | --- | --- | --- | --- | --- |
|  | **Intervention** N=144 | **Control**  N=178 | **Intervention**  N=103 | **Control**  N=178 | **Intervention** N=131 | **Control**  N=144 |
| **Grade**^1^ %: 6  7  8 | 35.9  33.8  30.3 | 34.3  34.9  30.9 | 36.9  29.1  34.0 | 32.4  32.4  35.2 | 35.1  35.9  29.0 | 27.8  32.6  39.6 |
| **Birth country parents** %:  Both NL  Both MR/TU  Both other  NL-MR/TU  NL-other  MR/TU-other | 12.8  33.3  34  9.9  5.0  5.0 | 8.2 40.6 28.8 11.2  8.2  2.9 | 9.0  38.0  31.0  8.0  7.0  7.0 | 8.0  39.4  31.4  14.3  3.4  3.4 | 8.4  36.6  32.1  9.2  8.4  5.3 | 9.4  33.1  38.1  15.8  0.7  2.9 |
| **Lives with** %:  Both parents together  Parents separated  Parent + new partner  Other | 82.5  9.1  7.0  1.4 | 77.3 14.2  6.3  2.3 | 80.0  17.0  1.0  2.0 | 77.8  15.9  4.0  2.3 | 77.1  13.7  4.6  4.6 | 73.2  19.7  4.2  2.8 |
| **Consumption soda** (mL/week) x͂ (IQR) | 750.0 (250.0-2547.0) | 580.0 (125.0-3240.0) | 500.0 (155.0-1500.0) | 580.0 (165.0-1830.0) | 540.0 (155.0-1740.0) | 540.0 (250.0-1740.0) |
| **Consumption energy/sports drinks** (mL/week) x͂ (IQR) | 125.0 (0.0-250.0) | 0.0 (0.0-250.0) | 125.0 (0.0-250.0) | 125.0 (0.0-281.3) | 125.0 (0.0-250.0) | 125.0 (0.0-375.0) |
| **Consumption candy** (portions/week) x͂ (IQR) | 4.0 (2.0-8.0) | 3.0 (1.5-6.0) | 4.0 (2.0-7.0) | 4.0 (2.0-7.0) | 3.5 (1.5-7.1) | 3.0 (1.5-7.0) |
| **Consumption snacks** (portions/week) x͂ (IQR) | 5.0 (2.5-12.0) | 4.0 (1.8-8.5) | 9.0 (2.5-15.3) | 6.8 (3.0-13.0) | 5.0 (2.5-10.5) | 5.0 (2.0-9.0) |
| **Active transport to school** (min) x̅ (SD) | 5.2 (4.7) | 6.3 (5.1) | 6.0 (4.7) | 7.4 (5.6) | 5.5 (5.2) | 7.5 (5.6) |
| **Outside play** (min/day) x̅ (SD) | 108.0 (66.5) | 84.0 (59.3) | 115.0 (60.5) | 92.8 (63.2) | 105.8 (58.5) | 92.9 (59.4) |
| **Sports participation** (min/day) x͂ (IQR) | 47.1 (25.7-102.9) | 34.3 (17.1-79.3) | 42.9 (21.4-85.7) | 38.6 (17.1-73.9) | 47.1 (24.6-78.2) | 38.6 (25.7-76.1) |
| **Watching TV/movies** (min/day) x̅ (SD) | 87.5 (63.6) | 88.6 (71.8) | 114.3 (92.5) | 108.7 (90.1) | 93.4 (72.7) | 97.9 (78.6) |
| **Gaming** (min/day) x̅ (SD) | 72.2 (73.0) | 90.5 (93.3) | 110.3 (87.2) | 95.0 (99.5) | 99.2 (91.0) | 105.9 (88.9) |
| **Self-rated health** (scale 0-100) x̅ (SD) | 82.9 (18.7) | 80.8 (21.2) | 82.1 (18.3) | 78.8 (21.3) | 78.2 (19.1) | 79.2 (20.9) |

*Notes.* mL=milliliter, min=minutes, MR=Morocco, NL=the Netherlands, TU=Turkey.
^1^ Mixed grades (such as 6/7) are added to the lowest grades.
