# Supplementary material for: How to Evaluate the Effectiveness of Health Promotion Actions Developed Through Youth-Centered Participatory Action Research
Source: Health Educ Behav. 2021 Oct 9;50(2):199–210. doi: 10.1177/10901981211046533 (PMC10021122; doi:10.1177/10901981211046533)
Supplement: sj-docx-4-heb-10.1177_10901981211046533 – Supplemental material for How to Evaluate the Effectiveness of Health Promotion Actions Developed Through Youth-Centered Participatory Action Research [file sj-docx-4-heb-10.1177_10901981211046533.docx]

**Online supp 4:** Characteristics of children with valid accelerometer data.

|  | **T0** | | **T1** | | **T2** | |
| --- | --- | --- | --- | --- | --- | --- |
|  | **Intervention** N=99 | **Control**  N=124 | **Intervention**  N=62 | **Control** N=87 | **Intervention** N=87 | **Control** N=77 |
| **Grade**^1^ (%): 6  7  8 | 31.6 35.7 32.7 | 32.0 36.1 32.0 | 43.5 29.0 27.4 | 22.1  41.9  36.0 | 42.5 37.9 19.5 | 32.5  42.9 24.7 |
| **Birth country parents** (%):  Both NL  Both MR/TU  Both other  NL-MR/TU  NL-other  MR/TU-other | 15.5 34.0 30.9 11.3 5.2 3.1 | 7.5 40.0 29.2 10.8 9.2 3.3 | 9.8 37.7 24.6 4.9 14.8 8.2 | 6.9 41.4 37.9 6.9 3.4 3.4 | 10.6 41.2 25.9 9.4 10.6 2.4 | 11.0 27.4 41.1 16.4 1.4 2.7 |
| **Lives with** (%):  Both parents together  Parents separate  Parent + new partner  Other | 80.8 10.1  8.1 1.0 | 77.2 13.8  6.5 2.4 | 85.2 8.2  3.3 3.3 | 80.5 11.5  4.6 3.4 | 81.2 9.4  5.9 3.5 | 66.7 28.0  5.3 0.0 |
| **Wear time** (min/day) x̅ (SD) | 759.8 (56.9) | 776.1 (42.5) | 766.9 (50.5) | 756.2 (58.0) | 773.4 (52.1) | 770.4 (51.3) |
| **Time spent sedentary** (min/day) x̅ (SD) | 440.0 (59.0) | 461.2 (57.0) | 433.3 (68.4) | 434.1 (67.6) | 447.7 (56.2) | 433.0 (60.8) |
| **Time spent in LPA** (min/day) x̅ (SD) | 261.8 (46.4) | 268.3 (64.2) | 263.9 (51.1) | 260.9 (51.8) | 265.5 (39.7) | 271.4 (42.5) |
| **Time spent in MVPA** (min/day) x̅ (SD) | 58.2 (21.0) | 50.7 (18.0) | 69.7 (31.3) | 60.9 (23.9) | 61.8 (23.0) | 66.0 (29.0) |
| **MVPA accumulated in bouts of ≥5 min** (min/day) x͂ (IQR) | 2.0 (0.0-3.9) | 2.4 (0.8-5.9) | 4.8 (1.4-8.1) | 2.7 (0.8-6.8) | 3.1 (1.9-6.6) | 4.2 (1.5-6.8) |
| **Sedentary time accumulated in bouts of ≥10 min** (min/day) x̅ (SD) | 99.4 (48.0) | 108.7 (49.2) | 101.6 (56.8) | 112.5 (54.1) | 101.6 (43.1) | 100.3 (43.3) |

*Notes.* LPA=light physical activity, min=minutes, MR=Morocco, MVPA=moderate-to-vigorous physical activity, NL=the Netherlands, TU=Turkey.
Only data is presented from outcome variables or relevant covariates.
^1^ Mixed grades (such as 6/7) are added to the lowest grades.
